# Cat Swarm Optimization Algorithm: A Survey and Performance Evaluation

**DOI:** 10.1155/2020/4854895

**Published:** 2020-01-22

**Authors:** Aram M. Ahmed, Tarik A. Rashid, Soran Ab. M. Saeed

**Affiliations:** ^1^International Academic Office, Kurdistan Institution for Strategic Studies and Scientific Research, Sulaymaniyah 46001, Iraq; ^2^Information Technology, Sulaimani Polytechnic University, Sulaymaniyah 46001, Iraq; ^3^Computer Science and Engineering, University of Kurdistan Hewler, Erbil 44001, Iraq

## Abstract

This paper presents an in-depth survey and performance evaluation of cat swarm optimization (CSO) algorithm. CSO is a robust and powerful metaheuristic swarm-based optimization approach that has received very positive feedback since its emergence. It has been tackling many optimization problems, and many variants of it have been introduced. However, the literature lacks a detailed survey or a performance evaluation in this regard. Therefore, this paper is an attempt to review all these works, including its developments and applications, and group them accordingly. In addition, CSO is tested on 23 classical benchmark functions and 10 modern benchmark functions (CEC 2019). The results are then compared against three novel and powerful optimization algorithms, namely, dragonfly algorithm (DA), butterfly optimization algorithm (BOA), and fitness dependent optimizer (FDO). These algorithms are then ranked according to Friedman test, and the results show that CSO ranks first on the whole. Finally, statistical approaches are employed to further confirm the outperformance of CSO algorithm.

## 1. Introduction

Optimization is the process by which the optimal solution is selected for a given problem among many alternative solutions. One key issue of this process is the immensity of the search space for many real-life problems, in which it is not feasible for all solutions to be checked in a reasonable time. Nature-inspired algorithms are stochastic methods, which are designed to tackle these types of optimization problems. They usually integrate some deterministic and randomness techniques together and then iteratively compare a number of solutions until a satisfactory one is found. These algorithms can be categorized into trajectory-based and population-based classes [1]. In trajectory-based types, such as a simulated annealing algorithm [2], only one agent is searching in the search space to find the optimal solution, whereas, in the population-based algorithms, also known as swarm Intelligence, such as particle swarm optimization (PSO) [3], multiple agents are searching and communicating with each other in a decentralized manner to find the optimal solution. Agents usually move in two phases, namely, exploration and exploitation. In the first one, they move on a global scale to find promising areas, while in the second one, they search locally to discover better solutions in those promising areas found so far. Having a trade-off between these two phases, in any algorithm, is very crucial because biasing towards either exploration or exploitation would degrade the overall performance and produce undesirable results [1]. Therefore, more than hundreds of swarm intelligence algorithms have been proposed by researchers to achieve this balance and provide better solutions for the existing optimization problems.

**Figure 1:**
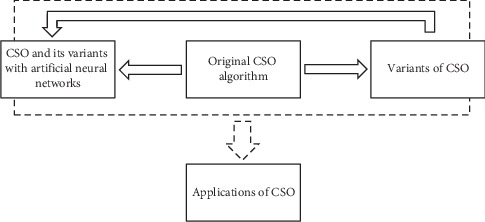
General framework for conducting the survey.

Cat swarm optimization (CSO) is a swarm Intelligence algorithm, which was originally invented by Chu et al. in 2006 [4, 5]. It is inspired by the natural behavior of cats, and it has a novel technique in modeling exploration and exploitation phases. It has been successfully applied in various optimization fields of science and engineering. However, the literature lacks a recent and detailed review of this algorithm. In addition, since 2006, CSO has not been compared against novel algorithms, i.e., it has been mostly compared with PSO algorithm while many new algorithms have been introduced since then. So, a question, which arises, is whether CSO competes with the novel algorithms or not? Therefore, experimenting CSO on a wider range of test functions and comparing it with new and robust algorithms will further reveal the potential of the algorithm. As a result, the aims of this paper are as follows: firstly, provide a comprehensive and detailed review of the state of art of CSO algorithm (see Figure 1), which shows the general framework for conducting the survey; secondly, evaluate the performance of CSO algorithm against modern metaheuristic algorithms. These should hugely help researchers to further work in the domain in terms of developments and applications.

The rest of the paper is organized as follows.  Section 2 presents the original algorithm and its mathematical modeling.  Section 3 is dedicated to reviewing all modified versions and variants of CSO.  Section 4 summarizes the hybridizing CSO algorithm with ANN and other non-metaheuristic methods.  Section 5 presents applications of the algorithm and groups them according to their disciplinary.  Section 6 provides performance evaluation, where CSO is compared against dragonfly algorithm (DA) [6], butterfly optimization algorithm (BOA) [7], and fitness dependent optimizer (FDO) [8]. Finally, Section 7 provides the conclusion and future directions.

## 2. Original Cat Swarm Optimization Algorithm

The original cat swarm optimization is a continuous and single-objective algorithm [4, 5]. It is inspired by resting and tracing behaviours of cats. Cats seem to be lazy and spend most of their time resting. However, during their rests, their consciousness is very high and they are very aware of what is happening around them. So, they are constantly observing the surroundings intelligently and deliberately and when they see a target, they start moving towards it quickly. Therefore, CSO algorithm is modeled based on combining these two main deportments of cats.

CSO algorithm is composed of two modes, namely, tracing and seeking modes. Each cat represents a solution set, which has its own position, a fitness value, and a flag. The position is made up of M dimensions in the search space, and each dimension has its own velocity; the fitness value depicts how well the solution set (cat) is; finally, the flag is to classify the cats into either seeking or tracing mode. Thus, we should first specify how many cats should be engaged in the iteration and run them through the algorithm. The best cat in each iteration is saved into memory, and the one at the final iteration will represent the final solution.

### 2.1. General Structure of the Algorithms

The algorithm takes the following steps in order to search for optimal solutions:

1. Specify the upper and lower bounds for the solution sets.
2. Randomly generate *N* cats (solution sets) and spread them in the *M* dimensional space in which each cat has a random velocity value not larger than a predefined maximum velocity value.
3. Randomly classify the cats into seeking and tracing modes according to MR. MR is a mixture ratio, which is chosen in the interval of [0, 1]. So, for example, if a number of cats *N* is equal to 10 and MR is set to 0.2, then 8 cats will be randomly chosen to go through seeking mode and the other 2 cats will go through tracing mode.
4. Evaluate the fitness value of all the cats according to the domain-specified fitness function. Next, the best cat is chosen and saved into memory.
5. The cats then move to either seeking or tracing mode.
6. After the cats go through seeking or tracing mode, for the next iteration, randomly redistribute the cats into seeking or tracing modes based on MR.
7. Check the termination condition; if satisfied; terminate the program; otherwise, repeat Step 4 to Step 6.

### 2.2. Seeking Mode

This mode imitates the resting behavior of cats, where four fundamental parameters play important roles: seeking memory pool (SMP), seeking range of the selected dimension (SRD), counts of dimension to change (CDC), and self-position considering (SPC). These values are all tuned and defined by the user through a trial-and-error method.

SMP specifies the size of seeking memory for cats, i.e., it defines number of candidate positions in which one of them is going to be chosen by the cat to go to, for example, if SMP was set to 5, then for each and every cat, 5 new random positions will be generated and one of them will be selected to be the next position of the cat. How to randomize the new positions will depend on the other two parameters that are CDC and SRD. CDC defines how many dimensions to be modified which is in the interval of [0, 1]. For example, if the search space has 5 dimensions and CDC is set to 0.2, then for each cat, four random dimensions out of the five need to be modified and the other one stays the same. SRD is the mutative ratio for the selected dimensions, i.e., it defines the amount of mutation and modifications for those dimensions that were selected by CDC. Finally, SPC is a Boolean value, which specifies whether the current position of a cat will be selected as a candidate position for the next iteration or not. So, for example, if the SPC flag is set to true, then for each cat, we need to generate (SMP-1) number of candidates instead of SMP number as the current position is considered as one of them. Seeking mode steps are as follows:

1. Make as many as SMP copies of the current position of Cat_*k*_.
2. For each copy, randomly select as many as CDC dimensions to be mutated. Moreover, randomly add or subtract SRD values from the current values, which replace the old positions as shown in the following equation:

(1) $$\begin{array}{c} Xjd_{\text{new}}=\left(1+\text{rand}*\text{SRD}\right)*Xjd_{\text{old}}, \end{array}$$

  where *Xjd*_old_ is the current position; *Xjd*_new_ is the next position; *j* denotes the number of a cat and *d* denotes the dimensions; and rand is a random number in the interval of [0, 1].
(3) Evaluate the fitness value (FS) for all the candidate positions.
(4) Based on probability, select one of the candidate points to be the next position for the cat where candidate points with higher FS have more chance to be selected as shown in equation (2). However, if all fitness values are equal, then set all the selecting probability of each candidate point to be 1.

(2) $$\begin{array}{c} \text{Pi}=\frac{\left|{\text{FS}}_{i}-{\text{FS}}_{b}\right|}{{\text{FS}}_{\max}-{\text{FS}}_{\min}},\;\text{where }0<i<j. \end{array}$$

If the objective is minimization, then FS_*b*_ = FS_max_; otherwise, FS_*b*_ = FS_min_.

### 2.3. Tracing Mode

This mode copies the tracing behavior of cats. For the first iteration, random velocity values are given to all dimensions of a cat's position. However, for later steps, velocity values need to be updated. Moving cats in this mode are as follows:

1. Update velocities (*V*_*k,d*_) for all dimensions according to equation (3).
2. If a velocity value outranged the maximum value, then it is equal to the maximum velocity.

(3) $$\begin{array}{c} V_{k,d}=V_{k,d}+r_{1}c_{1}\left(X_{\text{best},d}-X_{k,d}\right). \end{array}$$

(3) Update position of Cat_*k*_ according to the following equation:

(4) $$\begin{array}{c} X_{k,d}=X_{k,d}+V_{k,d}. \end{array}$$

**Figure 2:**
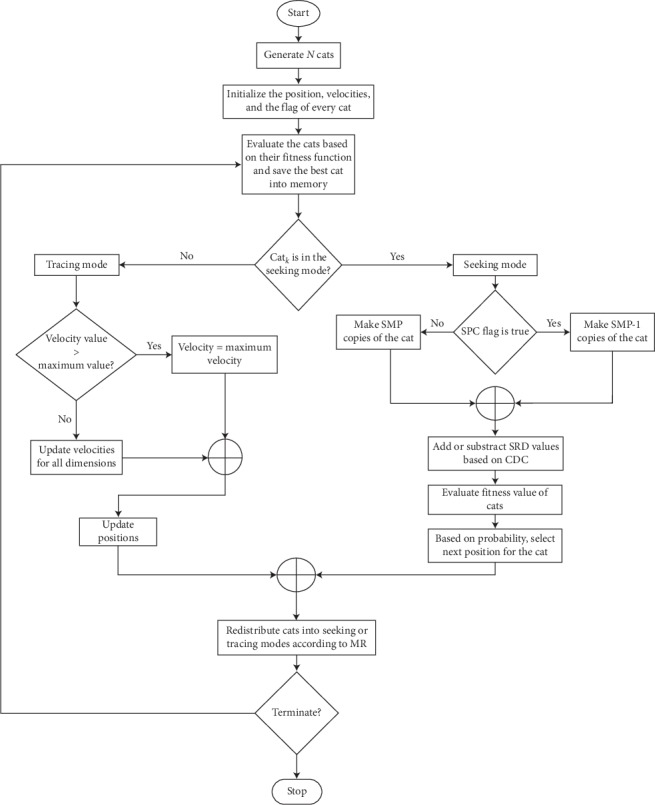
Cat swarm optimization algorithm general structure.

Refer to Figure 2 which recaps the whole algorithm in a diagram.

## 3. Variants of CSO

In the previous section, the original CSO was covered; this section briefly discusses all other variants of CSO found in the literature. Variants may include the following points: binary or multiobjective versions of the algorithm, changing parameters, altering steps, modifying the structure of the algorithm, or hybridizing it with other algorithms. Refer to Table 1, which presents a summary of these modifications and their results.

### 3.1. Discrete Binary Cat Swarm Optimization Algorithm (BCSO)

Sharafi et al. introduced the BCSO Algorithm, which is the binary version of CSO [9]. In the seeking mode, the SRD parameter has been substituted by another parameter called the probability of mutation operation (PMO). However, the proceeding steps of seeking mode and the other three parameters stay the same. Accordingly, the dimensions are selected using the CDC and then PMO will be applied. In the tracing mode, the calculations of velocity and position equations have also been changed into a new form, in which the new position vector is composed of binary digits taken from either current position vector or global position vector (best position vector). Two velocity vectors are also defined in order to decide which vector (current or global) to choose from.

### 3.2. Multiobjective Cat Swarm Optimization (MOCSO)

Pradhan and Panda proposed multiobjective cat swarm optimization (MOCSO) by extending CSO to deal with multiobjective problems [10]. MOCSO is combined with the concept of the external archive and Pareto dominance in order to handle the nondominated solutions.

### 3.3. Parallel Cat Swarm Optimization (PCSO)

Tsai and pan introduced parallel cat swarm optimization (PCSO) [11]. This algorithm improved the CSO algorithm by eliminating the worst solutions. To achieve this, they first distribute the cats into subgroups, i.e., subpopulations. Cats in the seeking mode move as they do in the original algorithm. However, in the tracing mode, for each subgroup, the best cat will be saved into memory and will be considered as the local best. Furthermore, cats move towards the local best rather than the global best. Then, in each group, the cats are sorted according to their fitness function from best to worst. This procedure will continue for a number of iterations, which is specified by a parameter called ECH (a threshold that defines when to exchange the information of groups). For example, if ECH was equal to 20, then once every 20 iterations, the subgroups exchange information where the worst cats will be replaced by a randomly chosen local best of another group. These modifications lead the algorithm to be computationally faster and show more accuracy when the number of iteration is fewer and the population size is small.

### 3.4. CSO Clustering

Santosa and Ningrum improved the CSO algorithm and applied it for clustering purposes [12]. The main goal was to use CSO to cluster the data and find the best cluster center. The modifications they did were two main points: firstly, removing the mixture ratio (MR) and hence forcing all the cats to go through both seeking and tracing mode. This is aimed at shortening the time required to find the best cluster center. Secondly, always setting the CDC value to be 100%, instead of 80% as in the original CSO, in order to change all dimensions of the candidate cats and increase diversity.

### 3.5. Enhanced Parallel Cat Swarm Optimization (EPCSO)

Tsai et al. further improved the PCSO Algorithm in terms of accuracy and performance by utilizing the orthogonal array of Taguchi method and called it enhanced parallel cat swarm optimization (EPCSO) [13]. Taguchi methods are statistical methods, which are invented by Japanese Engineer Genichi Taguchi. The idea is developed based on “ORTHOGONAL ARRAY” experiments, which improves the engineering productivity in the matters of cost, quality, and performance. In their proposed algorithm, the seeking mode of EPCSO is the same as the original CSO. However, the tracing mode has adopted the Taguchi orthogonal array. The aim of this is to improve the computational cost even when the number of agents increases. Therefore, two sets of candidate velocities will be created in the tracing mode. Then, based on the orthogonal array, the experiments will be run and accordingly the position of cats will be updated. Orouskhani et al. [14] added some partial modifications to EPCSO in order to further improve it and make it fit their application. The modifications were changing the representation of agents from the coordinate to a set; adding a newly defined cluster flag; and designing custom-made fitness function.

### 3.6. Average-Inertia Weighted CSO (AICSO)

Orouskhani et al. introduced an inertia value to the velocity equation in order to achieve a balance between exploration and exploitation phase. They experimented that (*w*) value is better to be selected in the range of [0.4, 0.9] where at the beginning of the operation, it is set to 0.9, and as the iteration number moves forward, (*w*) value gradually becomes smaller until it reaches 0.4 at the final iteration. Large values of (*w*) assist global search; whereas small values of (*w*) assist the local search. In addition to adding inertia value, the position equation was also reformed to a new one, in which averages of current and previous positions, as well as an average of current and previous velocities, were taken in the equation [14].

### 3.7. Adaptive Dynamic Cat Swarm Optimization (ADCSO)

Orouskhani et al. further enhanced the algorithm by introducing three main modifications [15]. Firstly, they introduced an adjustable inertia value to the velocity equation. This value gradually decreases as the dimension numbers increase. Therefore, it has the largest value for dimension one and vice versa. Secondly, they changed the constant (C) to an adjustable value. However, opposite to the inertia weight, it has the smallest value for dimension one and gradually increases until the final dimension where it has the largest value. Finally, they reformed the position equation by taking advantage of other dimensions' information.

### 3.8. Enhanced Hybrid Cat Swarm Optimization (Enhanced HCSO)

Hadi and Sabah proposed a hybrid system and called it enhanced HCSO [16, 17]. The goal was to decrease the computation cost of the block matching process in video editing. In their proposal, they utilized a fitness calculation strategy in seeking mode of the algorithm. The idea was to avoid calculating some areas by deciding whether or not to do the calculation or estimate the next search location to move to. In addition, they also introduced the inertia weight to the tracing mode.

### 3.9. Improvement Structure of Cat Swarm Optimization (ICSO)

Hadi and Sabah proposed combining two concepts together to improve the algorithm and named it ICSO. The first concept is parallel tracing mode and information exchanging, which was taken from PCSO. The second concept is the addition of an inertia weight to the position equation, which was taken from AICSO. They applied their algorithm for efficient motion estimation in block matching. Their goal was to enhance the performance and reduce the number of iterations without the degradation of the image quality [17].

### 3.10. Opposition-Based Learning-Improved CSO (OL-ICSO)

Kumar and Sahoo first proposed using Cauchy mutation operator to improve the exploration phase of the CSO algorithm in [34]. Then, they introduced two more modifications to further improve the algorithm and named it opposition-based learning-improved CSO (OL-ICSO). They improved the population diversity of the algorithm by adopting opposition-based learning method. Finally, two heuristic mechanisms (for both seeking and tracing mode) were introduced. The goal of introducing these two mechanisms was to improve the diverse nature of the populations and prevent the possibility of falling the algorithm into the local optima when the solution lies near the boundary of the datasets and data vectors cross the boundary constraints frequently [18].

### 3.11. Chaos Quantum-Behaved Cat Swarm Optimization (CQCSO)

Nie et al. improved the CSO algorithm in terms of accuracy and avoiding local optima trapping. They first introduced quantum-behaved cat swarm optimization (QCSO), which combined the CSO algorithm with quantum mechanics. Hence, the accuracy was improved and the algorithm avoided trapping in the local optima. Next, by incorporating a tent map technique, they proposed chaos quantum-behaved cat swarm optimization (CQCSO) algorithm. The idea of adding the tent map was to further improve the algorithm and again let the algorithm to jump out of the possible local optima points it might fall into [19].

### 3.12. Improved Cat Swarm Optimization (ICSO)

In the original algorithm, cats are randomly selected to either go into seeking mode or tracing mode using a parameter called MR. However, Kanwar et al. changed the seeking mode by forcing the current best cat in each iteration to move to the seeking mode. Moreover, in their problem domain, the decision variables are firm integers while solutions in the original cat are continuous. Therefore, from selecting the best cat, two more cats are produced by flooring and ceiling its value. After that, all probable combinations of cats are produced from these two cats [20].

### 3.13. Improved Cat Swarm Optimization (ICSO)

Kumar and Singh made two modifications to the improved CSO algorithm and called it ICSO [21]. They first improved the tracing mode by modifying the velocity and updating position equations. In the velocity equation, a random uniformly distributed vector and two adaptive parameters were added to tune global and local search movements. Secondly, a local search method was combined with the algorithm to prevent local optima problem.

### 3.14. Hybrid PCSOABC

Tsai et al. proposed a hybrid system by combining PCSO with ABC algorithms and named is hybrid PCSOABC [22]. The structure simply included running PCSO and ABC consecutively. Since PCSO performs faster with a small population size, the algorithm first starts with a small population and runs PCSO. After a predefined number of iterations, the population size will be increased and the ABC algorithm starts running. Since the proposed algorithm was simple and did not have any adjustable feedback parameters, it sometimes provided worse solutions than PCSO. Nevertheless, its convergence was faster than PCSO.

### 3.15. CSO-GA-PSOSVM

Vivek and Reddy proposed a new method by combining CSO with particle swarm intelligence (PSO), genetic algorithm (GA), and support vector machine (SVM) and called it CSO-GA-PSOSVM [23]. In their method, they adopted the GA mutation operator into the seeking mode of CSO in order to obtain divergence. In addition, they adopted all GA operators as well as PSO subtraction and addition operators into the tracing mode of CSO in order to obtain convergence. This hybrid metaheuristic system was then incorporated with the SVM classifier and applied on facial emotion recognition.

### 3.16. Hybrid CSO-Based Algorithm

Skoullis et al. introduced three modifications to the algorithm [24]. Firstly, they combined CSO with a local search refining procedure. Secondly, if the current cat is compared with the global best cat and their fitness values were the same, the global best cat will still be updated by the current cat. The aim of this is to achieve more diversity. Finally, cats are individually selected to go into either seeking mode or tracing mode.

### 3.17. Hybrid CSO-GA-SA

Sarswat et al. also proposed a hybrid system by combining CSO, GA, and SA and then incorporating it with a modularity-based method [25]. They named their algorithm hybrid CSO-GA-SA. The structure of the system was very simple and straight forward as it was composed of a sequential combination of CSO, GA, and SA. They applied the system to detect overlapping community structures and find near-optimal disjoint communities. Therefore, input datasets were firstly fed into CSO algorithm for a predefined number of iterations. The resulted cats were then converted into chromosomes and henceforth GA was applied on them. However, GA may fall into local optima, and to solve this issue, SA was applied afterward.

### 3.18. Modified Cat Swarm Optimization (MCSO)

Lin et al. combined a mutation operator as a local search procedure with CSO algorithm to find better solutions in the area of the global best [26]. It is then used to optimize the feature selection and parameters of the support vector machine. Additionally, Mohapatra et al. used the idea of using mutation operation before distributing the cats into seeking or tracing modes [27].

### 3.19. Normal Mutation Strategy-Based Cat Swarm Optimization (NMCSO)

Pappula et al. adopted a normal mutation technique to CSO algorithm in order to improve the exploration phase of the algorithm. They used sixteen benchmark functions to evaluate their proposed algorithm against CSO and PSO algorithms [28].

### 3.20. Improved Cat Swarm Optimization (ICSO)

Lin et al. improved the seeking mode of CSO algorithm. Firstly, they used crossover operation to generate candidate positions. Secondly, they changed the value of the new position so that SRD value and current position have no correlations [29]. It is worth mentioning that there are four versions of CSO referenced in [17, 20, 21, 29], all having the same name (ICSO). However, their structures are different.

### 3.21. Compact Cat Swarm Optimization (CCSO)

Zhao introduced a compact version of the CSO algorithm. A differential operator was used in the seeking mode of the proposed algorithm to replace the original mutation approach. In addition, a normal probability model was used in order to generate new individuals and denote a population of solutions [30].

### 3.22. Boolean Binary Cat Swarm Optimization (BBCSO)

Siqueira et al. worked on simplifying the binary version of CSO in order to increase its efficiency. They reduced the number of equations, replaced the continues operators with logic gates, and finally integrated the roulette wheel approach with the MR parameter [31].

### 3.23. Hybrid Cat Swarm Optimization-Crow Search (CSO-CS) Algorithm

Pratiwi proposed a hybrid system by combining CSO algorithm with crow search (CS) algorithm. The algorithm first runs CSO algorithm followed by the memory update technique of the CS algorithm and then new positions will be generated. She applied her algorithm on vehicle routing problem [32].

## 4. CSO and its Variants with Artificial Neural Networks

Artificial neural networks are computing systems, which have countless numbers of applications in various fields. Earlier neural networks were used to be trained by conventional methods, such as the backpropagation algorithm. However, current neural networks are trained by nature-inspired optimization algorithms. The training could be optimizing the node weights or even the network architectures [35]. CSO has also been extensively combined with neural networks in order to be applied in different application areas. This section briefly goes over those works, in which CSO is hybridized with ANN and similar methods.

### 4.1. CSO + ANN + OBD

Yusiong proposes combining ANN with CSO algorithm and optimal brain damage (OBD) approach. Firstly, the CSO algorithm is used as an optimization technique to train the ANN algorithm. Secondly, OBD is used as a pruning algorithm to decrease the complexity of ANN structure where less number of connections has been used. As a result, an artificial neural network was obtained that had less training errors and high classification accuracy [36].

### 4.2. ADCSO + GD + ANFIS

Orouskhani et al. combined ADCSO algorithm with gradient descent (GD) algorithm in order to tweak parameters of the adaptive network-based fuzzy inference system (ANFIS). In their method, the antecedent and consequent parameters of ANFIS were trained by CSO algorithm and GD algorithm consecutively [37].

### 4.3. CSO + SVM

Abed and Al-Asadi proposed a hybrid system based on SVM and CSO. The system was applied to electrocardiograms signals classification. They used CSO for the purpose of feature selection optimization and enhancing SVM parameters [38]. In addition, Lin et al. and Wang and Wu [39, 40] also combined CSO with SVM and applied it to a classroom response system.

### 4.4. CSO + WNN

Nanda proposed a hybrid system by combining wavelet neural network (WNN) and CSO algorithm. In their proposal, the CSO algorithm was used to train the weights of WNN in order to obtain the near-optimal weights [41].

### 4.5. BCSO + SVM

Mohamadeen et al. built a classification model based on BCSO and SVM and then applied it in a power system. The use of BCSO was to optimize SVM parameters [42].

### 4.6. CCSO + ANN

Wang et al. proposed designing an ANN that can handle randomness, fuzziness, and accumulative time effect in time series concurrently. In their work, the CSO algorithm was used to optimize the network structure and learning parameters at the same time [43].

### 4.7. CSO/PSO + ANN

Chittineni et al. used CSO and PSO algorithms to train ANN and then applied their method on stock market prediction. Their comparison results showed that CSO algorithm performed better than the PSO algorithm [44].

### 4.8. CS-FLANN

Kumar et al. combined the CSO algorithm with functional link artificial neural network (FLANN) to develop an evolutionary filter to remove Gaussian noise [45].

## 5. Applications of CSO

This section presents the applications of CSO algorithm, which are categorized into seven groups, namely, electrical engineering, computer vision, signal processing, system management and combinatorial optimization, wireless and WSN, petroleum engineering, and civil engineering. A summary of the purposes and results of these applications is provided in Table 2.

### 5.1. Electrical Engineering

CSO algorithm has been extensively applied in the electrical engineering field. Hwang et al. applied both CSO and PSO algorithms on an electrical payment system in order to minimize electricity costs for customers. Results indicated that CSO is more efficient and faster than PSO in finding the global best solution [46]. Economic load dispatch (ELD) and unit commitment (UC) are significant applications, in which the goal is to reduce the total cost of fuel is a power system. Hwang et al. applied the CSO algorithm on economic load dispatch (ELD) of wind and thermal generators [47]. Faraji et al. also proposed applying binary cat swarm optimization (BCSO) algorithm on UC and obtained better results compared to the previous approaches [48]. UPFC stands for unified power flow controller, which is an electrical device used in transmission systems to control both active and reactive power flows. Kumar and Kalavathi used CSO algorithm to optimize UPFC in order to improve the stability of the system [49]. Lenin and Reddy also applied ADCSO on reactive power dispatch problem with the aim to minimize active power loss [50]. Improving available transfer capability (ATC) is very significant in electrical engineering. Nireekshana et al. used CSO algorithm to regulate the position and control parameters of SVC and TCSC with the aim of maximizing power transfer transactions during normal and contingency cases [51]. The function of the transformers is to deliver electricity to consumers. Determining how reliable these transformers are in a power system is essential. Mohamadeen et al. proposed a classification model to classify the transformers according to their reliability status [42]. The model was built based on BCSO incorporation with SVM. The results are then compared with a similar model based on BPSO. It is shown that BCSO is more efficient in optimizing the SVM parameters. Wang et al. proposed designing an ANN that can handle randomness, fuzziness, and accumulative time effect in time series concurrently [43]. In their work, the CSO algorithm has been used to optimize the network structure and learning parameters at the same time. Then, the model was applied to two applications, which were individual household electric power consumption forecasting and Alkaline-surfactant-polymer (ASP) flooding oil recovery index forecasting in oilfield development. The current source inverter (CSI) is a conventional kind of power inverter topologies. Hosseinnia and Farsadi combined selective harmonic elimination (SHE) in corporation with CSO algorithm and then applied it on current source inverter (CSI) [52]. The role of the CSO algorithm was to optimize and tune the switching parameters and minimize total harmonic distortion. El-Ela et al. [53] used CSO and PCSO to find the optimal place and size of distributed generation units on distribution networks. Guo et al. [54] used MCSO algorithm to propose a novel maximum power point tracking (MPPT) approach to obtain global maximum power point (GMPP) tracking. Srivastava et al. used BCSO algorithm to optimize the location of phasor measurement units and reduce the required number of PMUs [55]. Guo et al. used CSO algorithm to identify the parameters of single and double diode models in solar cell models [56].

### 5.2. Computer Vision

Facial emotion recognition is a biometric approach to identify human emotion and classify them accordingly. Lin et al. and Wang and Wu [39, 40] proposed a classroom response system by combining the CSO algorithm with support vector machine to classify student's facial expressions. Vivek and Reddy also used CSO-GA-PSOSVM algorithm for the same purpose [23]. Block matching in video processing is computationally expensive and time consuming. Hadi and Sabah used CSO algorithm in block matching for efficient motion estimation [57]. The aim was to decrease the number of positions that needs to be calculated within the search window during the block matching process, i.e., to enhance the performance and reduce the number of iterations without the degradation of the image quality. The authors further improved their work and achieved better results by replacing the CSO algorithm with HCSO and ICSO in [16, 17], respectively. Kalaiselvan et al. and Lavanya and Natarajan [58, 59] used CSO Algorithm to retrieve watermarks similar to the original copy. In video processing, object tracking is the process of determining the position of a moving object over time using a camera. Hadi and Sabah used EHCSO in an object-tracking system for further enhancement in terms of efficiency and accuracy [60]. Yan et al. used BCSO as a band selection method for hyperspectral images [61]. In computer vision, image segmentation refers to the process of dividing an image into multiple parts. Ansar and Bhattacharya and Karakoyun et al. [62, 63] proposed using CSO algorithm incorporation with the concept of multilevel thresholding for image segmentation purposes. Zhang et al. combined wavelet entropy, ANN, and CSO algorithm to develop an alcohol use disorder (AUD) identification system [64]. Kumar et al. combined the CSO algorithm with functional link artificial neural network (FLANN) to remove the unwanted Gaussian noises from CT images [45]. Yang et al. combined CSO with L-BFGS-B technique to register nonrigid multimodal images [65]. Çam employed CSO algorithm to tune the parameters in the histogram stretching technique for the purpose of image enhancement [66].

### 5.3. Signal Processing

IIR filter stands for infinite impulse response. It is a discrete-time filter, which has applications in signal processing and communication. Panda et al. used CSO algorithm for IIR system identification [67]. The authors also applied CSO algorithm as an optimization mechanism to do direct and inverse modeling of linear and nonlinear plants [68]. Al-Asadi combined CSO Algorithm with SVM for electrocardiograms signal classification [38].

### 5.4. System Management and Combinatorial Optimization

In parallel computing, optimal task allocation is a key challenge. Shojaee et al. [69, 70] proposed using CSO algorithm to maximize system reliability. There are three basic scheduling problems, namely, open shop, job shop, and flow shop. These problems are classified as NP-hard and have many real-world applications. They coordinate assigning jobs to resources at particular times, where the objective is to minimize time consumption. However, their difference is mainly in having ordering constraints on operations. Bouzidi and Riffi applied the BCSO algorithm on job scheduling problem (JSSP) in [71]. They also made a comparative study between CSO and two other metaheuristic algorithms, namely, cuckoo search (CS) algorithm and the ant colony optimization (ACO) for JSSP in [72]. Then, they used the CSO algorithm to solve flow shop scheduling (FSSP) [73] and open shop scheduling problems (OSSP) as well [74]. Moreover, Dani et al. also applied CSO algorithm on JSSP in which they used a nonconventional approach to represent cat positions [75]. Maurya and Tripathi also applied CSO algorithm on bag-of-tasks and workflow scheduling problems in cloud systems [76]. Bouzidi and Riffi applied CSO algorithm on the traveling salesman problem (TSP) and the quadratic assignment problem (QAP), which are two combinatorial optimization problems [77]. Bouzidi et al. also made a comparative study between CSO algorithm, cuckoo search algorithm, and bat-inspired algorithm for addressing TSP [78]. In cloud computing, minimizing the total execution cost while allocating tasks to processing resources is a key problem. Bilgaiyan et al. applied CSO and MCSO algorithms on workflow scheduling in cloud systems [79]. In addition, Kumar et al. also applied BCSO on workflow scheduling in cloud systems [80]. Set cover problem (SCP) is considered as an NP-complete problem. Crawford et al. successfully applied the BCSO Algorithm to this problem [81]. They further improved this work by using Binarization techniques and selecting different parameters for each test example sets [82, 83]. Web services provide a standardized communication between applications over the web which have many important applications. However, discovering appropriate web services for a given task is challenging. Kotekar and Kamath used a CSO-based approach as a clustering algorithm to group service documents according to their functionality similarities [84]. Sarswat et al. applied Hybrid CSO-GA-SA to detect the overlapping community structures and find the near-optimal disjoint communities [25]. Optimizing the problem of controlling complex network systems is critical in many areas of science and engineering. Orouskhani et al. applied CSO algorithm to address a number of problems in optimal pinning controllability and thus optimized the network structure [85]. Skoullis et al. combined the CSO algorithm with local search refining procedure and applied it on high school timetabling problem [24]. Soto et al. combined BCSO with dynamic mixture ratios to organize the cells in manufacturing cell design problem [86]. Bahrami et al. applied a CSO algorithm on water resource management where the algorithm was used to find the optimal reservoir operation [87]. Kencana et al. used CSO algorithm to classify the feasibility of small loans in banking systems [88]. Majumder and Eldho combined the CSO algorithm with the analytic element method (AEM) and reverse particle tracking (RPT) to model novel groundwater management systems [89]. Rautray and Balabantaray used CSO algorithm to solve the multidocument summarization problem [90]. Thomas et al. combined radial point collocation meshfree (RPCM) approach with CSO algorithm to be used in the groundwater resource management [91]. Pratiwi created a hybrid system by combining the CSO algorithm and crow search (CS) algorithm and then used it to address the vehicle routing problem with time windows (VRPTW) [32]. Naem et al. proposed a modularity-based system by combining the CSO algorithm with K-median clustering technique to detect overlapping community in social networks [92].

### 5.5. Wireless and WSN

The ever-growing wireless devices push researchers to use electromagnetic spectrum bands more wisely. Cognitive radio (CR) is an effective dynamic spectrum allocation in which spectrums are dynamically assigned based on a specific time or location. Pradhan and Panda in [93, 94] combined MOCSO with fitness sharing and fuzzy mechanism and applied it on CR design. They also conducted a comparative analysis and proposed a generalized method to design a CR engine based on six evolutionary algorithms [95]. Wireless sensor network (WSN) refers to a group of nodes (wireless sensors) that form a network to monitor physical or environmental conditions. The gathered data need to be forwarded among the nodes and each node requires having a routing path. Kong et al. proposed applying enhanced parallel cat swarm optimization (EPCSO) algorithm in this area as a routing algorithm [33]. Another concern in the context of WSN is minimizing the total power consumption while satisfying the performance criteria. So, Tsiflikiotis and Goudos addressed this problem which is known as optimal power allocation problem, and for that, three metaheuristic algorithms were presented and compared [96]. Moreover, Pushpalatha and Kousalya applied CSO in WSN for optimizing cluster head selection which helps in energy saving and available bandwidth [97]. Alam et al. also applied CSO algorithm in a clustering-based method to handle channel allocation (CA) issue between secondary users with respect to practical constraints in the smart grid environment [98]. The authors of [99, 100] used the CSO algorithm to find the optimal location of sink nodes in WSN. Ram et al. applied CSO algorithm to minimize the sidelobe level of antenna arrays and enhance the directivity [101]. Ram et al. used CSO to optimize controlling parameters of linear antenna arrays and produce optimal designs [102]. Pappula and Ghosh also used Cauchy mutated CSO to make linear aperiodic arrays, where the goal was to reduce sidelobe level and control the null positions [103].

### 5.6. Petroleum Engineering

CSO algorithm has also been applied in the petroleum engineering field. For example, it was used as a good placement optimization approach by Chen et al. in [104, 105]. Furthermore, Wang et al. used CSO algorithm as an ASP flooding oil recovery index forecasting approach [43].

### 5.7. Civil Engineering

Ghadim et al. used CSO algorithm to create an identification model that detects early cracks in building structures [106].

## 6. Performance Evaluation

**Figure 3:**
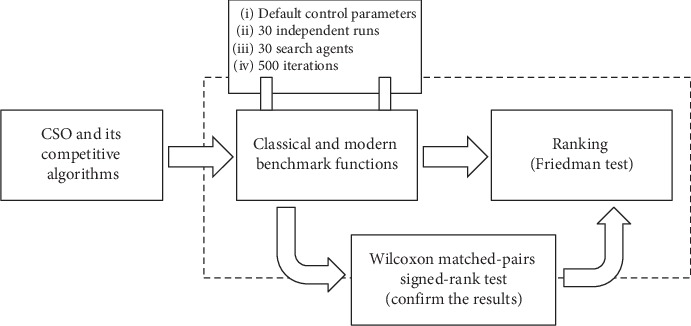
General framework of the performance evaluation process.

Many variants and applications of CSO algorithm were discussed in the above sections. However, benchmarking these versions and conducting a comparative analysis between them were not feasible in this work. This is because: firstly, their source codes were not available. Secondly, different test functions or datasets have been used during their experiments. In addition, since the emergence of CSO algorithm, many novel and powerful metaheuristic algorithms have been introduced. However, the literature lacks a comparative study between CSO algorithm and these new algorithms. Therefore, we conducted an experiment, in which the original CSO algorithm was compared against three new and robust algorithms, which were dragonfly algorithm (DA) [6], butterfly optimization algorithm (BOA) [7], and fitness dependent optimizer (FDO) [8]. For this, 23 traditional and 10 modern benchmark functions were used (see Figure 3), which illustrates the general framework for conducting the performance evaluation process. It is worth mentioning that for four test functions, BOA returned imaginary numbers and we set “N/A” for them.

### 6.1. Traditional Benchmark Functions

This group includes the unimodal and multimodal test functions. Unimodal test functions contain one single optimum while multimodal test functions contain multiple local optima and usually a single global optimum. F1 to F7 are unimodal test functions (Table 3), which are employed to experiment with the global search capability of the algorithms. Furthermore, F8 to F23 are multimodal test functions, which are employed to experiment with the local search capability of the algorithms. Refer to [107] for the detailed description of unimodal and multimodal functions.

### 6.2. Modern Benchmark Functions (CEC 2019)

These set of benchmark functions, also called composite benchmark functions, are complex and difficult to solve. The CEC01 to CEC10 functions as shown in Table 3 are of these types, which are shifted, rotated, expanded, and combined versions of traditional benchmark functions. Refer to [108] for the detailed description of modern benchmark functions.

The comparison results for CSO and other algorithms are given in Table 3 in the form of mean and standard deviations. For each test function, the algorithms are executed for 30 independent runs. For each run, 30 search agents were searching over the course of 500 iterations. Parameter settings are set as defaults for all algorithms, and nothing was changed.

**Figure 4:**
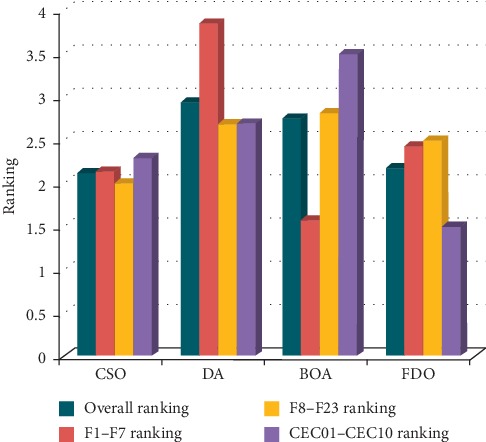
Ranking of algorithms according to different groups of test functions.

It can be noticed from Table 3 that the CSO algorithm is a competitive algorithm for the modern ones and provides very satisfactory results. In order to perceive the overall performance of the algorithms, they are ranked as shown in Table 4 according to different benchmark function groups. It can be seen that CSO ranks first in the overall ranking and multimodal test functions. Additionally, it ranks second in unimodal and CEC test functions (see Figure 4). These results indicate the effectiveness and robustness of the CSO algorithm. That being said, these results need to be confirmed statistically.  Table 5 presents the Wilcoxon matched-pairs signed-rank test for all test functions. In more than 85% of the results, *P* value is less than 0.05%, which proves that the results are significant and we can reject the null hypothesis that there is no difference between the means. It is worth mentioning that the performance of CSO can be further evaluated by comparing it against other new algorithms such as donkey and smuggler optimization algorithm [109], modified grey wolf optimizer [110], BSA and its variants [111], WOA and its variants [112], and other modified versions of DA [113].

## 7. Conclusion and Future Directions

Cat swarm optimization (CSO) is a metaheuristic optimization algorithm proposed originally by Chu et al. [5] in 2006. Henceforward, many modified versions and applications of it have been introduced. However, the literature lacks a detailed survey in this regard. Therefore, this paper firstly addressed this gap and presented a comprehensive review including its developments and applications.

CSO showed its ability in tackling different and complex problems in various areas. However, just like any other metaheuristic algorithm, CSO algorithm possesses strengths and weaknesses. The tracing mode resembles the global search process while the seeking mode resembles the local search process. This algorithm enjoys a significant property for which these two modes are separated and independent. This enables researchers to easily modify or improve these modes and hence achieve a proper balance between exploration and exploitation phases. In addition, fast convergence is another strong point of this algorithm, which makes it a sensible choice for those applications that require quick responses. However, the algorithm has a high chance of falling into local optima, known as premature convergence, which can be considered as the main drawback of the algorithm.

Another concern was the fact that CSO algorithm was not given a chance to be compared against new algorithms since it has been mostly measured up against PSO and GA algorithms in the literature. To address this, a performance evaluation was conducted to compare CSO against three new and robust algorithms. For this, 23 traditional benchmark functions and 10 modern benchmark functions were used. The results showed the outperformance of CSO algorithm, in which it ranked first in general. The significance of these results was also confirmed by statistical methods. This indicates that CSO is still a competitive algorithm in the field.

In the future, the algorithm can be improved in many aspects; for example, different techniques can be adapted to the tracing mode in order to solve the premature convergence problem or transforming MR parameter is static in the original version of CSO. Transforming this parameter into a dynamic parameter might improve the overall performance of the algorithm.

## Figures and Tables

**Table 1:** Summary of the modified versions of the CSO algorithm.

Comparison of	With	Testing field	Performance	Reference
CSO (original)	PSO and weighted-PSO	Six test functions	Better	[4, 5]
BCSO	GA, BPSO, and NBPSO	Four test functions (sphere, Rastrigin, Ackley, and Rosenbrock)	Better	[9]
MOCSO	NSGA-II	Cooperative spectrum sensing in cognitive radio	Better	[10]
PCSO	CSO and weighted-PSO	Three test functions (Rosenbrock, Rastrigrin, and Griewank)	Better when the number of iteration is fewer and the population size is small	[11]
CSO clustering	*K*-means and PSO clustering	Four different clustering datasets (Iris, Soybean, Glass, and Balance Scale)	More accurate but slower.	[12]
EPCSO	PCSO, PSO-LDIW, PSO-CREV, GCPSO, MPSO-TVAC, CPSO-H6, PSO-DVM	Five test functions and aircraft schedule recovery problem	Better	[13]
AICSO	CSO	Three test functions (Rastrigrin, Griewank, and Ackley)	Better	[14]
ADCSO	CSO	Six test functions (Rastrigrin, Griewank, Ackley, axis parallel, Trid10, and Zakharov)	Better except for Griewank test function.	[15]
Enhanced HCSO	PSO	Motion estimation block-matching	Better	[16, 17]
ICSO	PSO	Motion estimation block-matching	Better	[17]
OL-ICSO	*K*-median, PSO, CSO, and ICSO	ART1, ART2, Iris, CMC, Cancer, and Wine datasets	Better	[18]
CQCSO	QCSO, CSO, PSO, and CPSO	Five test functions (Schaffer, Shubert, Griewank, Rastrigrin, and Rosenbrock) and multipeak maximum power point tracking for a photovoltaic array under complex conditions	Better	[19]
ICSO	CSO and PSO	The 69-bus test distribution system	Better	[20]
ICSO	CSO, BCSO, AICSO, and EPCSO	Twelve test functions (sphere, Rosenbrock, Rastrigin, Griewank, Ackley, Step, Powell, Schwefel, Schaffer, Zakharov's, Michalewicz, quartic) and five real-life clustering problems (Iris, Cancer, CMC, Wine, and Glass)	Better	[21]
Hybrid PCSOABC	PCSO and ABC	Five test functions	Better	[22]
CSO-GA-PSO_SVM_	CSO + SVM (CSO_SVM_)	66 feature points from each face of CK + (Cohn Kanade) dataset	Better	[23]
Hybrid CSO-based algorithm	GA, EA, SA, PSO, and AFS	School timetabling test instances	Better	[24]
Hybrid CSO-GA-SA	SLPA and CFinder	Seven datasets (Karate, Dolphin, Polbooks, Football, Net-Science, Power, Indian Railway)	Better	[25]
MCSO	CSO	Nine datasets from UCI	Better	[26]
MCSO	CSO	Eight dataset	Better	[27]
NMCSO	CSO, PSO	Sixteen benchmark functions	Better	[28]
ICSO	CSO	Ten datasets from UCI	Better	[29]
cCSO	DE, PSO, CSO	47 benchmark functions	Better	[30]
BBCSO	Binary particle swarm optimization (BPSO), binary genetic algorithm (BGA), binary CSO	0/1 Knapsack optimization problem	Better	[31]
CSO-CS	N/A	VRP instances from http://neo.lcc.uma.es/vrp/	N/A	[32]

**Table 2:** The purposes and results of using CSO algorithm in various applications.

Purpose	Results	Ref.
CSO applied on electrical payment system in order to minimize electricity cost for customers	CSO outperformed PSO	[46]
CSO applied on economic load dispatch (ELD) of wind and thermal generator	CSO outperformed PSO	[47]
BCSO applied on unit commitment (UC)	CSO outperformed LR, ICGA, BF, MILP, ICA, and SFLA	[48]
Applied CSO algorithm on UPFC to increase the stability of the system	IEEE 6-bus and 14-bus networks were used in the simulation experiments and desirable results were achieved	[49]
Applied ADCSO on reactive power dispatch problem to minimize active power loss	IEEE 57-bus system was used in the simulation experiments, in which ADCSO outperformed 16 other optimization algorithms	[50]
Applied CSO algorithm to regulate the position and control parameters of SVC and TCSC to improve available transfer capability (ATC)	IEEE 14-bus and IEEE 24-bus systems were used in the simulation experiments, in which the system provided better results after adopting CSO	[51]
Building a classification model based on BCSO and SVM to classify the transformers according to their reliability status.	The model performed better compared to a similar model, which was based on BPSO and VSM	[42]
Applied CSO to optimize the network structure and learning parameters of an ANN model named CPNN-CSO, which is used to predict household electric power consumption	CPNN-CSO outperformed ANFIS and similar methods with no CSO such as PNN and CPNN	[43]
Applied CSO and selective harmonic elimination (SHE) algorithm on current source inverter (CSI)	CSO successfully optimized the switching parameters of CSI and hence minimized the total harmonic distortion	[52]
Applied both CSO, PCSO, PSO-CFA, and ACO-ABC on distributed generation units on distribution networks	IEEE 33-bus and IEEE 69-bus distribution systems were used in the simulation experiments and CSO outperformed the other algorithms	[53]
Applied MCSO on MPPT to achieve global maximum power point (GMPP) tracking	MCSO outperformed PSO, MPSO, DE, GA, and HC algorithms	[54]
Applied BCSO to optimize the location of phasor measurement units and reduce the required number of PMUs	IEEE 14-bus and IEEE 30-bus test systems were used in the simulation. BCSO outperformed BPSO, generalized integer linear programming, and effective data structure-based algorithm	[55]
Used CSO algorithm to identify the parameters of single and double diode models in solar cell system	CSO outperformed PSO, GA, SA, PS, Newton, HS, GGHS, IGHS, ABSO, DE, and LMSA	[56]
Applied CSO and SVM to classify students' facial expression	The results show 100% classification accuracy for the selected 9 face expressions	[39]
Applied CSO and SVM to classify students' facial expression	The system achieved satisfactory results	[40]
Applied CSO-GA-PSOSVM to classify students' facial expression	The system achieved 99% classification accuracy	[23]
Applied CSO, HCSO and ICSO in block matching for efficient motion estimation	The system reduced computational complexity and provided faster convergence	[16, 17, 57]
Used CSO algorithm to retrieve watermarks similar to the original copy	CSO outperformed PSO and PSO time-varying inertia weight factor algorithms	[58, 59]
Sabah used EHCSO in an object-tracking system to obtain further efficiency and accuracy	The system yielded desirable results in terms of efficiency and accuracy	[60]
Used BCSO as a band selection method for hyperspectral images	BCSO outperformed PSO	[61]
Used CSO and multilevel thresholding for image segmentation	CSO outperformed PSO	[62]
Used CSO and multilevel thresholding for image segmentation	PSO outperformed CSO	[63]
Used CSO, ANN and wavelet entropy to build an AUD identification system.	CSO outperformed GA, IGA, PSO, and CSPSO	[64]
Used CSO and FLANN to remove the unwanted Gaussian noises from CT images	The proposed system outperformed mean filter and adaptive Wiener filter.	[45]
Used CSO with L-BFGS-B technique to register nonrigid multimodal images	The system yielded satisfactory results	[65]
Used CSO in image enhancement to optimize parameters of the histogram stretching technique	PSO outperformed CSO	[66]
Used CSO algorithm for IIR system identification	CSO outperformed GA and PSO	[67]
Applied CSO to do direct and inverse modeling of linear and nonlinear plants	CSO outperformed GA and PSO	[68]
Used CSO and SVM for electrocardiograms signal classification	Optimizing SVM parameters using CSO improved the system in terms of accuracy	[38]
Applied CSO to increase reliability in a task allocation system	CSO outperformed GA and PSO	[69, 70]
Applied CSO on JSSP	The benchmark instances were taken from OR-Library. CSO yielded desirable results compared to the best recorded results in the dataset reference.	[71]
Applied BCSO on JSSP	ACO outperformed CSO and cuckoo search algorithms	[72]
Applied CSO on FSSP	Carlier, Heller, and Reeves benchmark instances were used, CSO can solve problems of up to 50 jobs accurately	[73]
Applied CSO on OSSP	CSO performs better than six metaheuristic algorithms in the literature.	[74]
Applied CSO on JSSP	CSO performs better than some conventional algorithms in terms of accuracy and speed.	[75]
Applied CSO on bag-of-tasks and workflow scheduling problems in cloud systems	CSO performs better than PSO and two other heuristic algorithms	[76]
Applied CSO on TSP and QAP	The benchmark instances were taken from TSPLIB and QAPLIB. The results show that CSO outperformed the best results recorded in those dataset references.	[77]
Comparison between CSO, cuckoo search, and bat-inspired algorithm to solve TSP problem	The benchmark instances are taken from STPLIB. The results show that CSO falls behind the other algorithms	[78]
Applied CSO and MCSO on workflow scheduling in cloud systems	CSO performs better than PSO	[79]
Applied BCSO on workflow scheduling in cloud systems	BCSO performs better than PSO and BPSO	[80]
Applied BCSO on SCP	BCSO performs better than ABC	[81]
Applied BCSO on SCP	BCSO performs better than binary teaching-learning-based optimization (BTLBO)	[82, 83]
Used a CSO as a clustering mechanism in web services.	CSO performs better than K-means	[84]
Applied hybrid CSO-GA-SA to find the overlapping community structures.	Very good results were achieved. Silhouette coefficient was used to verify these results in which was between 0.7 and 0.9	[25]
Used CSO to optimize the network structures for pinning control	CSO outperformed a number of heuristic methods	[85]
Applied CSO with local search refining procedure to address high school timetabling problem	CSO outperformed genetic algorithm (GA), evolutionary algorithm (EA), simulated annealing (SA), particle swarm optimization (PSO) and artificial fish swarm (AFS).	[24]
BCSO with dynamic mixture ratios to address the manufacturing cell design problem	BCSO can effectively tackle the MCDP problem regardless of the scale of the problem	[86]
Used CSO to find the optimal reservoir operation in water resource management	CSO outperformed GA	[87]
Applied CSO to classify the the feasibility of small loans in banking systems	CSO resulted in 76% of accuracy in comparison to 64% resulted from OLR procedure.	[88]
Used CSO, AEM and RPT to build a groundwater management systems	CSO outperformed a number of metaheuristic algorithms in addressing groundwater management problem	[89]
Applied CSO to solve the multidocument summarization problem	CSO outperformed harmonic search (HS) and PSO	[90]
Used CSO and (RPCM) to address groundwater resource management	CSO outperformed a similar model based on PSO	[91]
Applied CSO-CS to solve VRPTW	CSO-CS successfully solves the VRPTW problem. The results show that the algorithm convergences faster by increasing population and decreasing *cdc* parameter.	[32]
Applied CSO and K-median to detect overlapping community in social networks	CSO and K-median provides better modularity than similar models based on PSO and BAT algorithm	[92]
Applied MOCSO, fitness sharing, and fuzzy mechanism on CR design	MOCSO outperformed MOPSO, NSGA-II and MOBFO	[93, 94]
Applied CSO and five other metaheuristic algorithms to design a CR engine	CSO outperformed the GA, PSO, DE, BFO and ABC algorithms	[95]
Applied EPCSO on WSN to be used as a routing algorithm	EPCSO outperformed AODV, a ladder diffusion using ACO and a ladder diffusion using CSO.	[33]
Applied CSO on WSN in order to solve optimal power allocation problem	PSO is marginally better for small networks. However, CSO outperformed PSO and cuckoo search algorithm	[96]
Applied CSO on WSN to optimize cluster head selection	The proposed system outperformed the existing systems by 75%.	[97]
Applied CSO on CR based smart grid communication network to optimize channel allocation	The proposed system obtains desirable results for both fairness-based and priority-based cases	[98]
Applied CSO in WSN to detect optimal location of sink nodes	CSO outperformed PSO in reducing total power consumption.	[99, 100]
Applied CSO on time modulated concentric circular antenna array to minimize the sidelobe level of antenna arrays and enhance the directivity	CSO outperformed RGA, PSO and DE algorithms	[101]
Applied CSO to optimize the radiation pattern controlling parameters for linear antenna arrays.	CSO successfully tunes the parameters and provides optimal designs of linear antenna arrays.	[102]
Applied Cauchy mutated CSO to make linear aperiodic arrays, where the goal was to reduce sidelobe level and control the null positions	The proposed system outperformed both CSO and PSO	[103]
Applied CSO and analytical formula-based objective function to optimize well placements	CSO outperformed DE algorithm	[104]
Applied CSO to optimize well placements considering oilfield constraints during development.	CSO outperformed GA and DE algorithms	[105]
CSO applied to optimize the network structure and learning parameters of an ANN model, which is used to predict an ASP flooding oil recovery index	The system successfully forecast the ASP flooding oil recovery index	[42]
Applied CSO to build an identification model to detect early cracks in beam type structures	CSO yields a desirable accuracy in detecting early cracks	[106]

**Table 3:** Comparison results of CSO algorithm with modern metaheuristic algorithms.

	CSO		DA		BOA		FDO		*f*_min_
Functions	AV	STD	AV	STD	AV	STD	AV	STD	
F1	3.50*E* − 14	6.34*E* − 14	15.24805	23.78914	1.01*E* − 11	1.66*E* − 12	2.13*E* − 23	1.06*E* − 22	0
F2	2.68*E* − 08	2.61*E* − 08	1.458012	0.869819	4.65*E* − 09	4.63*E* − 10	0.047175	0.188922	0
F3	7.17*E* − 09	1.16*E* − 08	136.259	151.9406	1.08*E* − 11	1.71*E* − 12	2.39*E* − 06	1.28*E* − 05	0
F4	0.010352	0.007956	3.262584	2.112636	5.25*E* − 09	5.53*E* − 10	4.93*E* − 08	9.09*E* − 08	0
F5	8.587858	0.598892	374.9048	691.5889	8.935518	0.02146	21.58376	39.66721	0
F6	1.151759	0.431511	12.07847	17.97414	1.04685	0.346543	7.15*E* − 22	2.80*E* − 21	0
F7	0.026026	0.015039	0.035679	0.023538	0.001513	0.00056	0.612389	0.299315	0
F8	−2855.11	359.1697	−2814.14	432.944	NA	NA	−10502.1	15188.77	−418.9829 × 5
F9	24.01772	6.480946	26.53478	11.20011	28.6796	20.17813	7.940883	4.110302	0
F10	3.754226	1.680534	2.827344	1.042434	3.00*E* − 09	1.16*E* − 09	7.76*E* − 15	2.46*E* − 15	0
F11	0.355631	0.19145	0.680359	0.353454	1.35*E* − 13	6.27*E* − 14	0.175694	0.148586	0
F12	1.900773	1.379549	2.083215	1.436402	0.130733	0.084891	7.737715	4.714534	0
F13	1.160662	0.53832	1.072302	1.327413	0.451355	0.138253	4.724571	6.448214	0
F14	0.998004	3.39*E* − 07	1.064272	0.252193	1.52699	0.841504	2.448453	1.766953	1
F15	0.001079	0.00117	0.005567	0.012211	0.000427	9.87*E* − 05	0.001492	0.003609	0.00030
F16	−1.03162	1.53*E* − 05	−1.03163	4.76*E* − 07	NA	NA	−1.00442	0.149011	−1.0316
F17	0.304253	1.81*E* − 06	0.304251	0	0.310807	0.004984	0.397887	5.17*E* − 15	0.398
F18	3.003667	0.004338	3.000003	1.22*E* − 05	3.126995	0.211554	3	2.37*E* − 07	3
F19	−3.8625	0.00063	−3.86262	0.00037	NA	NA	−3.86015	0.003777	−3.86
F20	−3.30564	0.045254	−3.25226	0.069341	NA	NA	−3.06154	0.380813	−3.32
F21	−9.88163	0.90859	−7.28362	2.790655	−4.44409	0.383552	−4.19074	2.664305	−10.1532
F22	−10.2995	0.094999	−8.37454	2.726577	−4.1496	0.715469	−4.89633	3.085016	−10.4028
F23	−10.0356	1.375583	−6.40669	2.892797	−4.12367	0.859409	−4.03276	2.517357	−10.5363
CEC01	1.58*E* + 09	1.71*E* + 09	3.8*E* + 10	4.03*E* + 10	58930.69	11445.72	4585.278	20707.63	1
CEC02	19.70367	0.580672	83.73248	100.1326	18.91597	0.291311	4	3.28*E* − 09	1
CEC03	13.70241	2.35*E* − 06	13.70263	0.000673	13.70321	0.000617	13.7024	1.68*E* − 11	1
CEC04	179.1984	55.37322	371.2471	420.2062	20941.5	7707.688	33.08378	16.81143	1
CEC05	2.671378	0.171923	2.571134	0.304055	6.176949	0.708134	2.13924	0.087218	1
CEC06	11.21251	0.708359	10.34469	1.335367	11.83069	0.771166	12.13326	0.610499	1
CEC07	365.2358	164.997	534.3862	240.0417	1043.895	215.3575	120.4858	13.82608	1
CEC08	5.499615	0.484645	5.86374	0.51577	6.337199	0.359203	6.102152	0.769938	1
CEC09	6.325862	1.295848	8.501541	16.90603	2270.616	811.4442	2	2.00*E* − 10	1
CEC10	21.36829	0.06897	21.29284	0.176811	21.4936	0.079492	2.718282	4.52*E* − 16	1

**Table 4:** Ranking of CSO algorithm compared to the modern metaheuristic algorithms.

Test functions	Ranking of CSO	Ranking of DA	Ranking of BOA	Ranking of FDO
F1	2	4	3	1
F2	2	4	1	3
F3	2	4	1	3
F4	3	4	1	2
F5	1	4	2	3
F6	3	4	2	1
F7	2	3	1	4
F8	2	3	4	1
F9	2	3	4	1
F10	4	3	2	1
F11	3	4	1	2
F12	2	3	1	4
F13	3	2	1	4
F14	1	2	3	4
F15	2	4	1	3
F16	1	2	4	3
F17	3	4	2	1
F18	3	2	4	1
F19	2	3	4	1
F20	1	2	4	3
F21	1	2	3	4
F22	1	2	4	3
F23	1	2	3	4
Cec01	3	4	2	1
Cec02	3	4	2	1
Cec03	2	3	4	1
Cec04	2	3	4	1
Cec05	3	2	4	1
Cec06	2	1	3	4
Cec07	2	3	4	1
Cec08	1	2	4	3
Cec09	2	3	4	1
Cec10	3	2	4	1
Total	70	97	91	72
Overall ranking	**2.121212**	2.939394	2.757576	2.181818
F1–F7 subtotal	15	27	**11**	17
F1–F7 ranking	2.142857	3.857143	**1.571429**	2.428571
F8–F23 subtotal	**32**	43	45	40
F8–F23 ranking	**2**	2.6875	2.8125	2.5
CEC01–CEC10 subtotal	23	27	35	**15**
CEC01–CEC10 ranking	2.3	2.7	3.5	**1.5**

**Table 5:** Wilcoxon matched-pairs signed-rank test.

Test functions	CSO vs. DA	CSO vs. BOA	CSO vs. FDO
F1	<0.0001	<0.0001	<0.0001
F2	<0.0001	<0.0001	0.0003
F3	<0.0001	<0.0001	0.2286
F4	<0.0001	<0.0001	<0.0001
F5	<0.0001	0.0879	0.0732
F6	0.0008	0.271	<0.0001
F7	0.077	<0.0001	<0.0001
F8	0.586	N/A	<0.0001
F9	0.2312	0.3818	<0.0001
F10	0.0105	<0.0001	<0.0001
F11	<0.0001	<0.0001	0.0002
F12	0.4	<0.0001	<0.0001
F13	<0.0001	<0.0001	0.0185
F14	0.4	<0.0001	0.0003
F15	0.0032	0.0004	0.9515
F16	<0.0001	N/A	<0.0001
F17	<0.0001	<0.0001	<0.0001
F18	<0.0001	<0.0001	<0.0001
F19	0.2109	N/A	0.6554
F20	0.0065	N/A	<0.0001
F21	0.0057	<0.0001	<0.0001
F22	0.1716	<0.0001	<0.0001
F23	<0.0001	<0.0001	<0.0001
cec01	<0.0001	<0.0001	<0.0001
cec02	0.001	<0.0001	<0.0001
cec03	0.0102	<0.0001	<0.0001
cec04	0.0034	<0.0001	<0.0001
cec05	0.1106	<0.0001	<0.0001
cec06	0.0039	0.0007	<0.0001
cec07	0.0002	<0.0001	<0.0001
cec08	0.0083	<0.0001	<0.0001
cec09	0.115	<0.0001	<0.0001
cec10	0.0475	<0.0001	<0.0001
